# An antibody developability triaging pipeline exploiting protein language models

**DOI:** 10.1080/19420862.2025.2472009

**Published:** 2025-03-04

**Authors:** James Sweet-Jones, Andrew C.R. Martin

**Affiliations:** Institute of Structural and Molecular Biology, Division of Biosciences, University College London, London, UK

**Keywords:** Antibodies, developability, machine learning, prediction, protein language models

## Abstract

Therapeutic monoclonal antibodies (mAbs) are a successful class of biologic drugs that are frequently selected from phage display libraries and transgenic mice that produce fully human antibodies. However, binding affinity to the correct epitope is necessary, but not sufficient, for a mAb to have therapeutic potential. Sequence and structural features affect the developability of an antibody, which influences its ability to be produced at scale and enter trials, or can cause late-stage failures. Using data on paired human antibody sequences, we introduce a pipeline using a machine learning approach that exploits protein language models to identify antibodies which cluster with antibodies that have entered the clinic and are therefore expected to have developability features similar to clinically acceptable antibodies, and triage out those without these features. We propose this pipeline as a useful tool in candidate selection from large libraries, reducing the cost of exploration of the antibody space, and pursuing new therapeutics.

## Introduction

Monoclonal antibodies (mAbs) have been shown to be a successful class of biologic drugs which have potential to treat a wide variety of diseases owing to their ability to target a specific antigen, and therefore potentially any step in a disease pathway.^1,2^ As of early 2025, at least 130 mAbs have received regulatory approval from the U.S. Food and Drug Administration or the European Medicines Agency (db.antibodysociety.org/) with at least 42 being considered as ‘fully-human’, either from transgenic mice, phage display libraries, or cloned from recovering patients.^3–5^ The annual growth of this sector has increased by between 20% and 30% per year^6,7^ and is likely to continue to grow as interest increases in the use of antibodies to target previously undruggable targets.^8^ Despite this, throughout the clinical pipeline for the development of new mAbs, there is a high risk of failure, causing costly discontinuation from trials.^9^

Simultaneously, efforts in single-cell sequencing techniques have been applied to understand how the antibody repertoire functions and changes over time at the level of single B cells.^10–13^ This has given researchers the ability to generate dense digital libraries of paired variable heavy (V_H_) and variable light (V_L_) human antibody sequences that vastly outnumber previous databases resulting from sequence or structural data (KabatMan,^14^ IMGT,^15^ SAbDAb^16^ AbDb^17^ and EMBLIg (abybank.org/emblig/)). Online repositories including the Observed Antibody Space (OAS),^18^ cAb-Rep^19^ and BRepertoire^20^ allow researchers access to these resources.

With the generation of these *in silico* databases, efforts to develop screening statistics to identify sequences with physical characteristics similar to approved therapeutics has become a driver in the field. Usually, these have been based on antibody developability, which is loosely defined as an antibody’s intrinsic ability to be produced on an industrial scale, to maintain reasonable stability in long-term storage and in patients, and to be safely tolerated by the patient.^21,22^ Such considerations have now become important in the early stages of drug screening to select the best quality candidates and avoid costly late-stage failures.^1^ Furthermore, developability is important, but does not guarantee success in clinical trials, where candidates may face discontinuation for safety or efficacy reasons. Identifying factors important in determining success in clinical trials has also eluded researchers.

Physicochemical features, including surface charged patches, surface hydrophobic patches, low thermostability, and post-translational modification sites that introduce heterogeneity, have become associated with poor antibody developability.^23^ Those features that compromise the stability of the antibody can cause unfolding, increase the propensity to aggregate in solution and can increase immunogenicity.^24,25^ At the lead candidate stage, well-defined experimental assays for measurement are important in the selection of a final lead.^26,27^ However, it has become useful to predict these features at an earlier stage using computational means. To this end, sequence-based statistics have been developed based on these features and are available for use in drug discovery pipelines, including the Developability Index,^28,29^ AbPred,^30^ and, more recently, the Therapeutic Antibody Profiler (TAP)^31^ and Therapeutic Antibody Developability Analysis (TA-DA Score).^32^ However, these tools can fall short in identifying leads from large libraries of data, requiring computationally expensive 3D modeling, or only taking one antibody at a time, which is usually expected already to be a potential lead candidate.

In order to take advantage of the wealth of data now available, the field has also turned to machine learning as a new avenue of exploration.^1,33,34^ For protein sequences to be suitable inputs for machine learning problems, it is necessary to encode them numerically. Previously, this has been done by using evolutionary or physicochemical and structural features,^35–37^ and simple regression models to identify features of high importance, or to predict features from the sequence as done in AbPred.^30^ Negron et al.^32^ expanded on this work and identified previously mentioned characteristics, including hydrophobicity (assessed by hydrophobic interaction chromatography), thermostability (Tm, assessed by differential scanning fluorimetry) and aggregation (assessed by cross-interaction chromatography) that were associated with the identification of clinically acceptable mAbs. Furthermore, this work has demonstrated an ability to separate clinical antibody sequences from antibody repertoires and to assign a developability score based on these features as part of their TA-DA score.

Many studies, including those described above^28–32^ and others^38^ as well as reviews,^39–41^ have described the importance of predicting developability and most of these approaches rely on the assumption that clinical antibodies (i.e., approved, discontinued and in-development mAbs) have a range of properties related to developability such that novel antibodies with similar (predicted) properties will also be clinically acceptable. This is not to say that antibodies with very different properties will necessarily fail in the clinic, but that such approaches allow one to focus on antibodies most likely to succeed. The need to exploit ‘big data’ and artificial intelligence in the development of biologics has been discussed by Fernández-Quintero et al.^39^ and Narayanan et al.^42^ A newer method of encoding protein sequences is to use ‘protein language models’.^43^ These are deep learning encoders trained on the relationships between residues in a sequence using millions of sequences. The results give dense numerical representations of sequences that may then be used as training data for machine learning models and, over the last few years, have revolutionized predictive methods in all areas of bioinformatics (see Lin et al.,^44^ for example). Their power comes from their ability to encode more information, including less obvious features and combinatorial or multi-factor features (e.g., from interaction of amino acids).

In this study, we hypothesized that, rather than directly predicting physical properties related to developability, antibodies with developable traits may be selected by encoding them using protein language models and comparing the encoded antibodies with encoded sequences of current clinical mAbs. Thus, our approach is, in principle, similar to many other approaches (including TAP), that look for similarity in properties to clinical antibodies, but, unlike these methods, we do not explicitly predict developability features, but instead exploit the power of protein language models for encoding antibody sequences.

Our goal is then to build a high-throughput triaging pipeline exploiting preliminary simple physicochemical screening followed by machine learning using protein language models which may be used to select antibodies most likely to have good developability characteristics from large libraries.

## Results

### Simple physicochemical properties of clinical and library antibodies

As a first step, we looked at using physicochemical properties to attempt to identify antibodies with clinically acceptable properties in a set of library antibodies. The aim was to see whether the clinical mAbs have a restricted distribution of these properties compared with antibodies from a library, similar to the approach used by Raybould *et al*,^31^ except here, we use only sequence statistics that can be calculated quickly without high computational expense.

A dataset was collated consisting of paired V_H_ and V_L_ sequences of clinical stage human mAbs (*n=*144) from the October 2021 release of TheraSabDab^3^ marked as ‘Whole mAb’ (Supplementary Table S1) and 10,000 paired sequences randomly selected from the OAS online repertoire repository (accessed January 2022)^18^ (Supplementary Table S2). We refer to this set of sequences from OAS as our ‘library’.

Physicochemical properties, including predicted *ΔG* of unfolding,^45^ iso-electric point (pI)^46^ and CDR-H3 loop length^47^ were calculated. Using a Mann-Whitney U test, we observed that there were statistical differences in the CDR-H3 length and in the predicted *ΔG* of unfolding for concatenated V_H_ and V_L_ chains between therapeutic and library antibodies (Table 1 and Supplementary Table S3). While this demonstrates a difference between human repertoire antibodies and what is found in the clinical mAb dataset, the mean values are relatively similar in the two datasets, making it difficult to use this as an approach to identify antibodies with clinically acceptable developability characteristics, although it can be used to reject clear outliers.

**Table 1.** Means and standard deviation of sequence-calculated physicochemical properties for fully human mAb therapeutics (*n=*144) and repertoire human antibodies from OAS (*n=*10,000, the ‘Human Library Antibodies’). p-values were calculated using a Mann-Whitney U test.

Feature	Human Therapeutic mAbs	Human LibraryAntibodies	p-value
CDR-H3 Loop Length	12.1 ± 6.65	15.0 ± 10.54	0.00049
*ΔG* V_H_ (kJ mol^−1^)	7614 ± 3260	6583 ± 3441	0.00014
*ΔG* V_L_ (kJ mol^−1^)	1086 ± 2381	796 ± 2614	0.14
Concatenated V_H_/V_L_ *ΔG* (kJ mol^−1^)	9248 ± 3896	7944 ± 4238	0.00015
Mean pI of V_H_/V_L_	7.9 ± 1.30	7.8 ± 1.24	0.025

### Identifying clinical-like antibodies from repertoires using unsupervised learning

An unsupervised learning model was proposed as an approach to identify library antibodies with clinical-antibody-like properties. Just as with approaches such as TAP, we hypothesize that clinical mAbs (which have probably undergone some developability assessment) should cluster in some *N*-dimensional space and that repertoire antibodies with similar properties would be positioned close to the clinical mAbs. To train an unsupervised learning method, the library and clinical V_H_ and V_L_ sequences were padded according to the Chothia numbering scheme, then independently encoded with various language models: ESM,^44^ AbLang,^48^ Sapiens^49^ and AntiBERTy.^50^ The encodings generated 130,048 features per paired V_H_/V_L_ sequence. All language models had a similar performance for this task, with AntiBERTy somewhat out-performing the other methods (data not shown).

**Figure 1.**
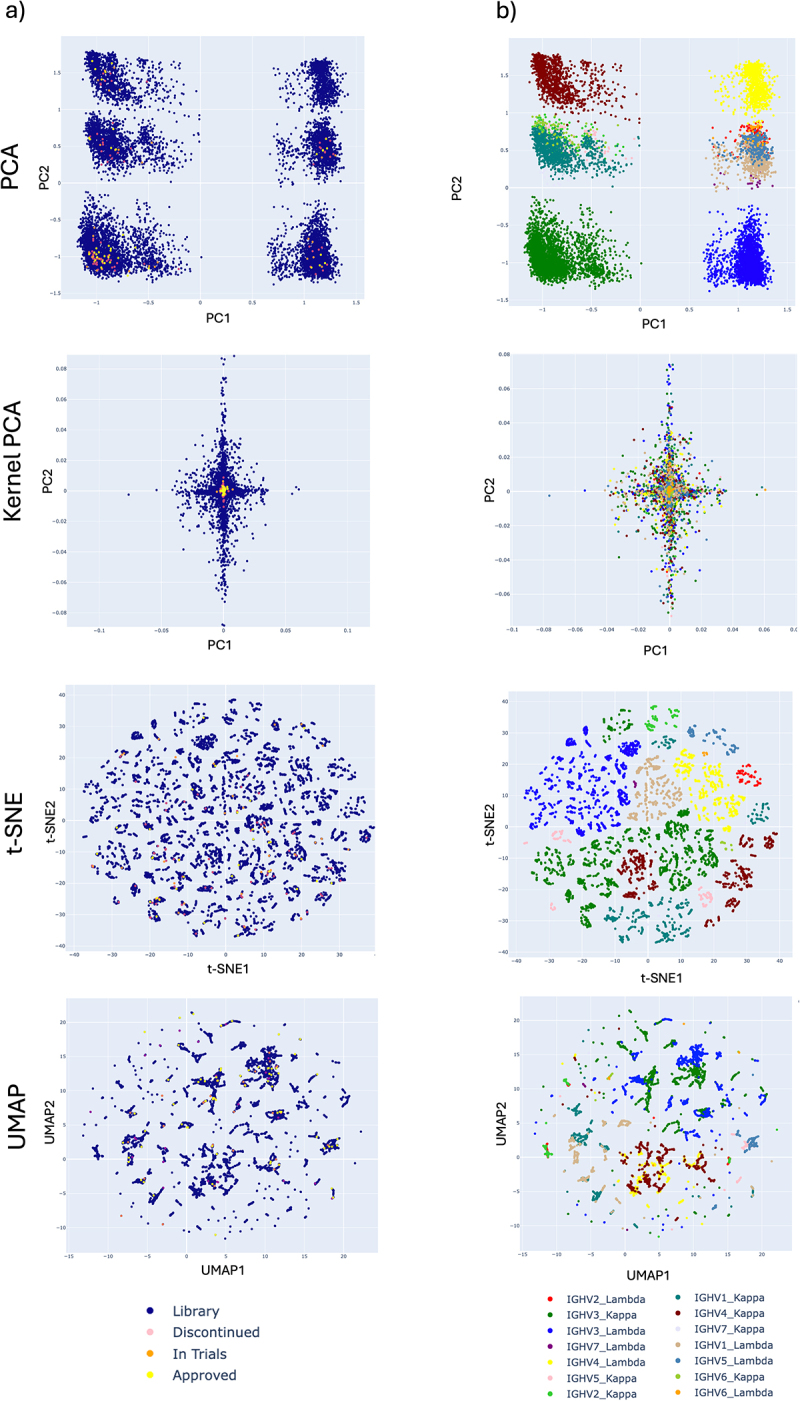
Scatter plots of unsupervised machine learning models trained on clinical (*n=*144) and library (*n=*10,000) paired antibody sequences encoded with the AntiBERTy protein language model. Plots are color coded by a) clinical stage or b) heavy chain V region germline gene and light chain type (λ or κ).

**Figure 2.**
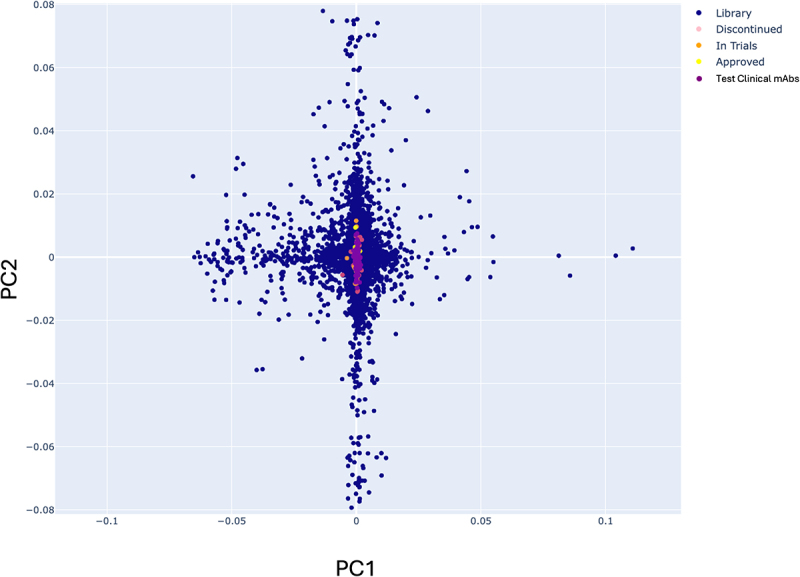
Scatter plot of kernel PCA (kernel=‘rbf’, γ = 500) clinical mAbs trained on clinical (*n=*144), library (*n=*10,000) and a held-back test set of clinical (*n=*203) paired human antibody sequences encoded with the AntiBERTy language model.

Various unsupervised machine learning models were tested: linear Principal Component Analysis (PCA),^51^ kernel PCA,^51^ 2-dimensional (2D) ‘t-distributed Stochastic Neighbor Embedding’ (t-SNE)^52^ and ‘Uniform Manifold Approximation and Projection’ (UMAP)^53^ (Figure 1a). These algorithms demonstrate how library antibodies are positioned against clinical mAbs also encoded with the AntiBERTy language model. For the linear PCA, t-SNE or UMAP, data were arranged into discrete groups of antibodies which are dictated by V_H_ and V_L_ gene germline pairing (Figure 1b). However, kernel PCA with a radial basis kernel function (γ = 500, Supplementary Figure S1), when viewing the first two principal components, gave a useful pattern of clustering where library antibodies form a radial pattern with clinical mAbs positioned around the origin (Figure 1a). This was also true of a held-back dataset of human-derived clinical mAbs (*n=*203) named with the 2016 and 2022 naming conventions^54^ in which the source infix (‘-u-’ for human or ‘-zu-’ for humanized) was removed, and therefore human mAbs could not be identified using the ‘−umab’ but not ‘−zumab’ approach used to identify human mAbs with the earlier naming schemes (see Methods and Supplementary Table S4). These held-back antibodies were positioned close to the original dataset of human-clinical mAbs (Figure 2). This led us to conclude that repertoire antibodies which are positioned close to clinical mAbs may be likely to share the developability properties necessary and should be taken forward for potential development.

**Figure 3.**
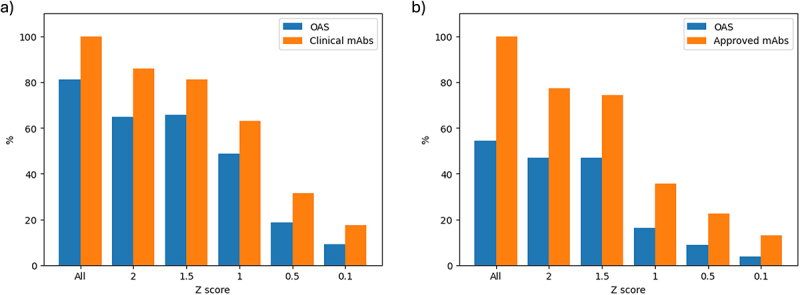
Percentages of OAS (library) and a) human clinical mAbs of any developmental stage, or b) only those with market approval, captured by the ellipse function drawn from the distribution of clinical mAbs. Z-scores denote how wide the distributions for the major axis of ellipse may be drawn with ‘All’ representing a Z-score selected such that all of the clinical (or approved, respectively) antibodies are captured.

Cutoffs were then established to select the repertoire antibodies which cluster with the clinical mAbs in order to extract them. An ellipse function was used in which the principal component with the greater range for clinical mAbs was taken to be the major axis, and the lesser range as the minor axis. Z-score thresholds (the number of standard deviations away from the mean) along the two principal components of the clinical mAbs were used to select where the extremes of the ellipse should be placed. The Z-score thresholds were optimized by measuring the proportion of the clinical mAbs captured by the ellipse against the proportion of the library antibodies also captured in the same ellipse. It was expected that, since the spread of antibodies was even across the first two principal components of the kernel PCA, roughly equal proportions of both groups would be captured. This was done using all human clinical mAbs (Figure 3a) and with only approved human mAbs (Figure 3b).

Comparing Figures 3a and 3b it can be seen that the bars for the OAS (library) antibodies are consistently lower when the Z-scores are based on the approved antibodies than they are when based on the clinical (i.e., approved, discontinued and in-development) antibodies. This indicates that the approved antibodies occupy a tighter distribution than the clinical antibodies. While it is obvious that the approved antibodies will be a subset of the clinical antibodies, it is less obvious that they will form a tighter cluster in this projection of the AntiBERTy-encoded parameter space. This led us to conclude that there may be characteristics of the approved antibodies identified by the protein language model that would allow them to be separated from the antibodies that were discontinued.

### Using supervised machine learning to distinguish approved and discontinued clinical antibodies

It is evident that having suitable developability profiles alone is not sufficient for an antibody to succeed in clinical trials and the clinical dataset used to identify library antibodies with properties similar to clinical mAbs contained discontinued antibodies. There are many reasons why an antibody could fail in clinical trials. Some of these are intrinsic to the sequence (e.g., immunogenicity, developability), while others are target-specific (e.g., binding affinity, nature of the epitope, on- or off-target side-effects).^1,9,55^ It is also likely that the effectiveness threshold for a drug to be taken forward from Phase 3 trials will be higher if there are already effective drugs on the market. However, given the differences observed above, we considered it worthwhile to attempt to train a predictor that might be able to identify drug-like antibodies that are more likely to succeed in the clinic. We assembled a dataset of the V_H_ and V_L_ amino acid sequences for 115 approved and 150 discontinued antibodies from the TheraSabDab^3^ (Supplementary Table S5).

Unlike the comparison of human clinical mAbs and library antibodies, which had statistical differences in their physicochemical properties (Table 1), there were no statistically significant differences in any of the basic physicochemical properties between approved and discontinued antibodies using a Mann-Whitney U test. This included the G score^56^ used as a method for predicting immunogenicity (Table 2). The largest quantitative difference was that the discontinued antibodies had a lower mean length for the CDR-H3 loop (Supplementary Table S6). The lack of statistically significant differences is perhaps not surprising given that both approved and discontinued antibodies will almost certainly have undergone a developability assessment and possibly optimization before entering clinical trials. It was also seen that the approved and discontinued groups had similar proportions of V_H_ and V_L_ V-gene germline pairings (Suplementary Figure S2).

**Table 2.** Means of sequence-calculated physicochemical properties for all market approved and discontinued mAbs (including human, humanized, chimeric and murine). p-values were calculated using a Mann-Whitney U test.

Feature	Approved	Discontinued	p-value
CDR-H3 Loop Length	13.4 ± 4.25	10.7 ± 3.36	0.17
V_H_ *ΔG* (kJ mol^−1^)	7008 ± 3806	7592 ± 3424	0.35
V_L_ *ΔG* (kJ mol^−1^)	2411 ± 1351	2546 ± 2675	0.33
Concatenated V_H_/V_L_ (kJ mol^−1^)	8434 ± 4855	1071 ± 4094	0.49
Mean pI of V_H_/V_L_	8.3 ± 1.18	7.9 ± 1.21	0.30
Mean Minimum G score	−1.0 ± 1.22	−0.8 ± 1.06	0.23

Details of the G score are given in Thullier *et al*.^56^.

As before, the V_H_ and V_L_ sequences for each antibody were padded and aligned using the Chothia numbering scheme and the sequences were then encoded with a selection of general protein (ESM^44^) and antibody-specific (AntiBERTy,^50^ AbLang,^48^ Sapiens^49^) protein language models. The encodings of the paired V_H_ and V_L_ sequences were concatenated and treated as a single set of data points per antibody. The encoded antibody sequences were used to train a selection of 15 supervised machine learning classifiers (see the Methods and Supplementary File: Supplementary ML.pdf). Models were trained with ten-fold cross validation (CV) and model performance was evaluated using the mean Matthews’ Correlation Coefficient (MCC)^57^ of the predictions of the test split of each fold. F-regression was used as a method of feature selection, by selecting the *k* features most correlated with the labels where *k* was set to [1,10,50,100,500,1000,2500,5000,10000].

**Figure 4.**
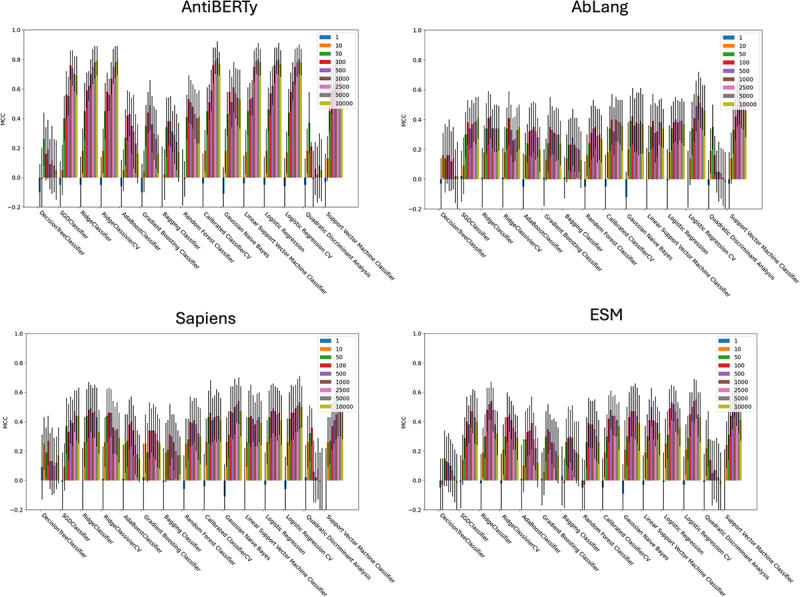
Matthews’ correlation coefficient (MCC) and standard deviation from ten-fold cross validation of 15 binary machine learning predictors classifying approved (*n=*115) and discontinued (*n=*150) therapeutic antibodies and encoded using four protein language models.

Generally performance across all classifiers was good (Supplementary Tables S7–10), but overall the best performance was obtained for the AntiBERTy encodings, particularly when using F-regression for feature selection with *k* set to 2500. The top-performing models were the Linear Support Vector Machine classifier (LinearSVC; MCC = 0.8 ± 0.08), Ridge Classifier (MCC = 0.78 ± 0.12) and Logistic regression (MCC = 0.80 ± 0.1) (See Figure 4). All methods were evaluated using a standard classification threshold of 0.5. The LinearSVC model was selected as the best model with a mean sensitivity of 0.86 ± 0.10 and specificity of 0.93 ± 0.05 across the 10 CV splits. In an attempt to improve the specificity further, this model was also assessed using a higher prediction threshold of 0.8. As expected, this resulted in a loss in sensitivity and an increase in specificity (Sn = 0.57 ± 0.17, Sp = 0.99 ± 0.03). This was accompanied by a decreased, but still respectable, MCC (0.64 ± 0.11). See Table 3 and Supplementary Figure S3, which shows confusion matrices for the raw outputs of this model at both probabilities.

**Table 3.** Summary performance of the LinearSVC supervised machine learning predictor for success in clinical trials.

	Prediction Threshold	Performance		
		MCC	Sensitivity	Specificity
Cross-validation	0.5	0.80 ± 0.08	0.86 ± 0.10	0.93 ± 0.05
	0.8	0.64 ± 0.11	0.57 ± 0.17	0.99 ± 0.03
Independent	0.5	0.14	0.50	0.64
	0.8	0.51	0.40	1.00

**Figure 5.**
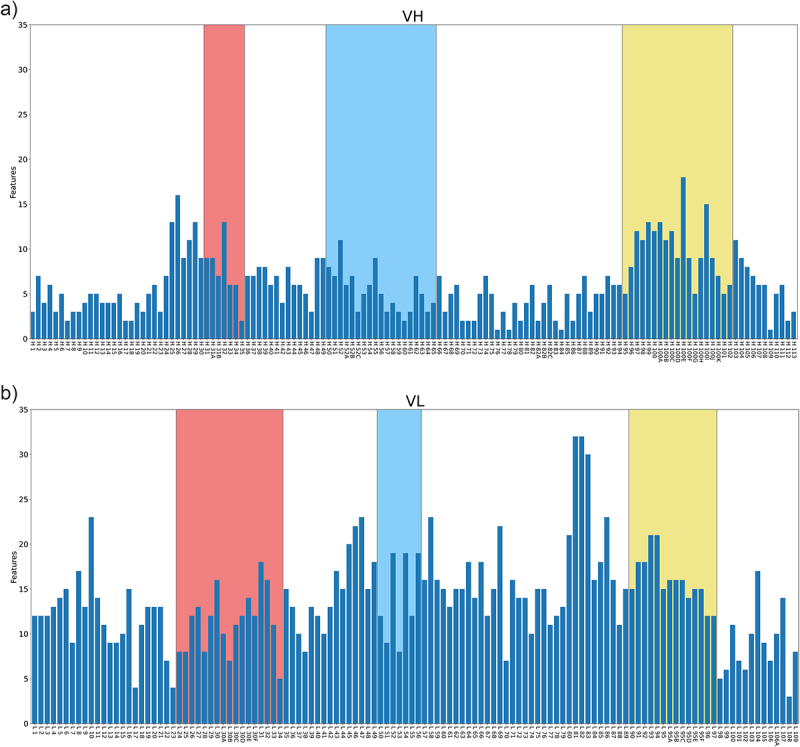
Locations of the top *k* features selected by F-regression from V_H_ and V_L_ chains of approved and discontinued mAbs encoded with the AntiBERTy language model^50^ where *k* = 2500. CDR loops (Kabat definition) are highlighted in red (CDR1), Blue (CDR2) and Yellow (CDR3) with Chothia numbering.

The location of the majority of selected features in the V_H_ or V_L_ sequences was then identified. Intuitively, it was expected that CDR-H3 would contain a high proportion of features, but this was not the case. Instead, a region in Framework 3 of the V_L_ chain had a high concentration of selected features, indicating that this region is highly important in how all the models have learned to distinguish these groups (Figure 5).

However, when a ‘held back’ dataset of therapeutics which have been approved (*n=*10) and discontinued (*n=*11) since the original access of TheraSabDab (Supplementary Table S11), the predictive score was found to be MCC = 0.14 using a prediction threshold of 0.5, but this increased to MCC = 0.51 with the higher prediction threshold of 0.8 (Supplementary Figure S4a). A summary of results for the cross-validation and independent test sets is shown in Table 3. On the independent test set, the default prediction threshold results in a reasonable sensitivity and specificity as a result of numerous false positives; increasing the threshold to 0.8 improves the specificity accompanied by a small decrease in sensitivity, resulting in a much improved MCC. Supplementary Figure S4b provides confusion matrices, MCC, sensitivity and specificity at prediction thresholds between 0.5 and 0.9.

That such good performance was achieved using basic predictive models was surprising. Because approved and discontinued antibodies did not show a statistically significant difference in features, including isoelectric point, thermostability and CDR-H3 length (Table 2), other properties, such as immunogenicity^25^ or the ability to access their targets, could be responsible for this ability to discriminate between these groups.^50^ It is also possible that subtle differences in V-region germline family pairing are involved, but Supplementary Figure S2 shows that these are broadly similar between the two groups.

### Assembling the pipeline to optimize performance and offer additional triaging

The kernel PCA model (γ = 500) shown to separate library antibodies which are positioned close to clinical mAbs, and the LinearSVC model using 2500 features shown to separate approved and discontinued antibodies using the AntiBERTy language model encodings, were used to build a pipeline capable of selecting developable antibodies from an input library. The encoding only needs to take place once and can be carried forward between the two layers of models: unsupervised and supervised, respectively.

**Figure 6.**
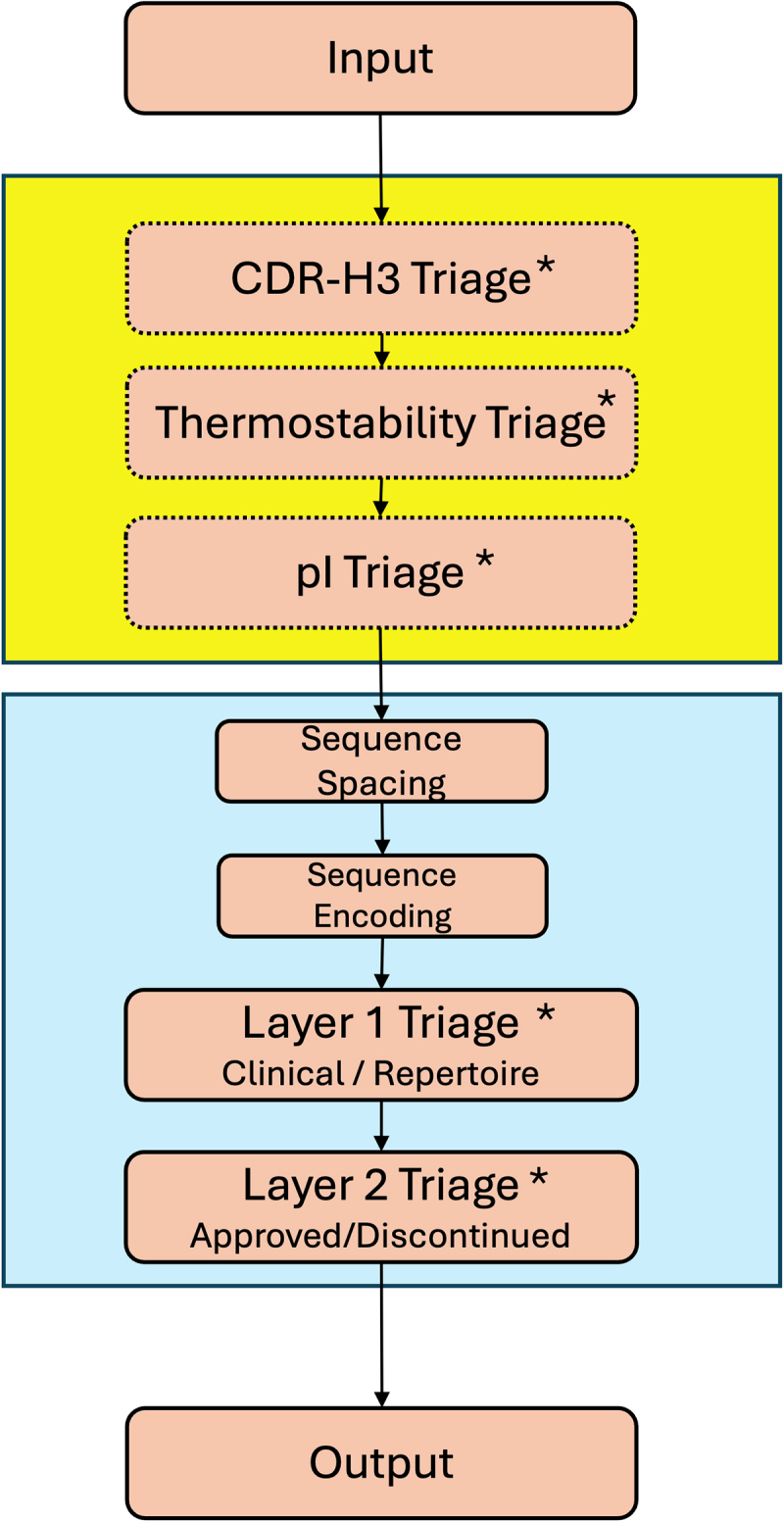
Schematic of the antibody triaging pipeline. The yellow box indicates optional physicochemical feature triaging steps calculating CDR-H3 length using AbNum.^58^ Thermostability (ΔG of unfolding) is calculated using the Oobatake Method^45^ and pI using the IPC method.^46^ the blue box indicates machine learning elements including spacing and encoding, as well as ‘Layer 1’ triage which is based on the kernel PCA model for separating antibodies with similar properties to clinical mAbs from the repertoire. The selection of antibodies to take forward is made using the ellipse function. ‘Layer 2’ is the supervised LinearSVC model trained to distinguish approved and discontinued clinical mAbs. ‘*’ indicates stages where stringency can be adjusted using Z-score thresholds, or the prediction threshold in the case of ‘Layer 2’.

The complete pipeline attempts first to remove antibodies with obvious developability issues through physicochemical properties using Z-scores taken from the values of the approved antibody dataset (‘Physicochemical Filtering’ as given in Table 1, default Z = 2); this saves computational time in numbering and encoding antibodies with the language model, as well as producing a better quality output. Antibodies with features typical of clinical mAbs are then selected from the unsupervised clustering of encoded antibodies using the ellipse function (‘Layer 1’) and are then classified according to whether they are likely to pass clinical trials (‘Layer 2’). A user may enter a library of human antibodies and obtain entries from those that are most likely to be successful. A schematic of the pipeline is shown in Figure 6 where stringency may be altered at each triaging step.

### Testing on an example dataset demonstrates points of parameter tuning for optimized output

To illustrate the application of our pipeline, a library of 10,382 paired B-cell receptor (BCR) sequences taken from six healthy blood donors^59^ was used as an example test dataset.

After physicochemical triaging, ‘Layer 1’ filtering is performed by performing PCA on the test data together with our ‘library’ antibodies from OAS and the previously used clinical dataset. The Z-score cutoffs are then calculated from the clinical dataset and the ellipse is generated and used to select antibodies from the test dataset.

**Figure 7.**
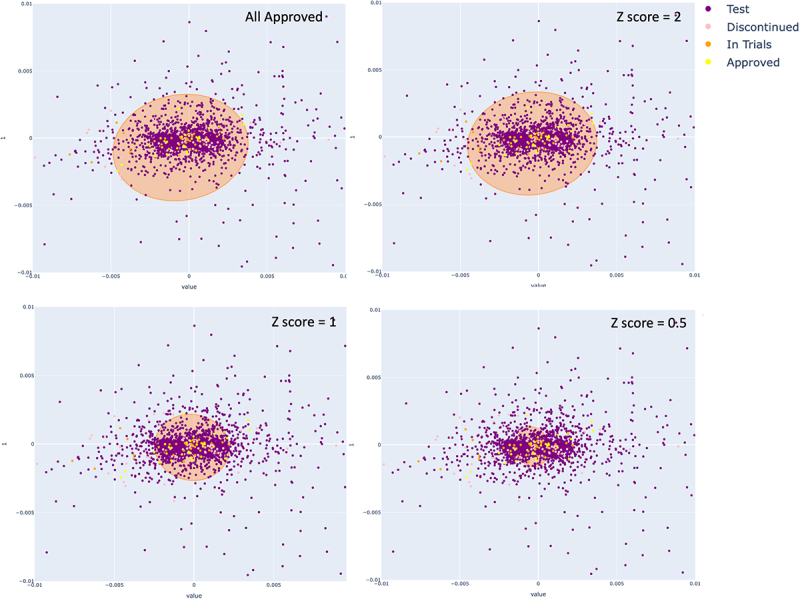
Scatter plots of clinical (*n=*144) and library (*n=*2740) paired antibody sequences encoded with the AntiBERTy protein language model and that have undergone dimensionality reduction using kernel Principal component analysis with a radial basis kernel function (gamma = 500). Different Z-scores of the distribution of clinical antibodies along PC1 are used as the extremes of the major axis to draw the ellipse function.

Using decreasing Z-scores for the physicochemical triaging of the sequences reduced the number of antibodies entering ‘Layer 1’ (Table 4 and Supplementary Table S11). Similarly, decreasing the Z-score of the ellipse function in ‘Layer 1’ generally reduces the number of sequences taken forward to ‘Layer 2’ (Figure 7). However, since the clustering is performed and the ellipse is recalculated for each dataset, there is some variation and, in one case (Table 4, no physicochemical filtering, ‘Layer 1’, Z-score = 1.0), there is a small rise in the number of antibodies compared with Z-score = 2.0. Increasing the prediction threshold used in ‘Layer 2’ also reduces the final number of selected antibodies.

**Table 4.** Number of antibodies from the test BCR library output from the triaging pipeline given different parameters of physicochemical filtering (PCF) and ‘Layer 1’ thresholds. For comparison, the minimum and mean TAP scores are provided, together with the percentage of negative TAP scores, after ‘Layer 1’ and ‘Layer 2’ shown separated by a ‘/’.

PCF Z-score		Layer 1 Filtering Z-Score			
		None	2.0	1.0	0.5
None	PCF Only	10492	–	–	–
	Layer 1	9875	8165	8186	6107
	Layer 2	3587	2981	2978	2232
	Min TAP Score	−110/-110	−110/-110	−110/-110	−110/-110
	Mean TAP Score	−18.58/-18.86	−18.52/-18.91	−18.53/-18.97	−18.28/-18.83
	% TAP Scores < 0	40.3/50.4	48.0/50.4	30.4/50.2	22.2/50.7
2.0	PCF Only	8045	–	–	–
	Layer 1	7508	7333	5855	3753
	Layer 2	2571	2514	1981	1272
	Min TAP Score	−110/-110	−110/-110	−110/-110	−110/-90
	Mean TAP Score	−18.07/-18.40	−18.05/-18.47	−18.12/-18.50	−18.11/-17.58
	% TAP Scores < 0	44.2/47.1	44.1/47.2	43.9/46.8	19.7/47.4
1.0	PCF Only	2740	–	–	–
	Layer 1	2359	2329	2056	1086
	Layer 2	808	797	705	361
	Min TAP Score	−90/-40	−90/-40	−90/-40	−90/-40
	Mean TAP Score	−16.77/-57	−16.78/-16.43	−16.83/-16.33	−16.99/-16.23
	% TAP Scores < 0	31.7/35.3	31.7/35.5	32.3/35.6	31.8/36.0
0.5	PCF Only	386	–	–	–
	Layer 1	308	231	157	39
	Layer 2	113	80	57	14
	Min TAP Score	−40/-40	−40/-40	−30/-30	−20/-20
	Mean TAP Score	−15.68/-15.0	−15.25/-5.38	−15.33/-15.00	−11.0/-12.5
	% TAP Scores < 0	25.3/31.9	21.5/32.5	28.7/38.6	35.6/38.6

As a comparison for the quality of antibodies output by the model, we checked the TAP score^31^ for each antibody from the test BCR library. The TAP score is a developability score where an antibody with values for selected physicochemical properties that are seen within the clinical mAb dataset are given a perfect score of 0, and antibodies with increasing numbers of ‘amber penalties’ where the values are at the extremes of, or outside (‘red flags’), the observed ranges are given negative scores. This is an indicator of developability, not whether an antibody is likely to be approved. It should be noted that, while the aims are broadly the same, this is a very different approach from our unsupervised machine learning: TAP relies solely on calculated or predicted physicochemical properties, while we use a subset of these properties only for a preliminary screen before using a clustering in high-dimensional space obtained from a protein language model.

The median TAP score for antibodies in the Test BCR library was 0, which means that more than half of these antibodies were predicted to have no developability warnings or red flags. However, the minimum TAP score observed from the library was −110, indicating there are antibodies in the library with many developability warnings or red flags. From the data in Table 4, it is clear that setting the physicochemical property filtering (PCF) in our approach to a more stringent Z-score (e.g., Z = 0.5) had the major effect in removing antibodies with the most negative TAP scores from the output. Indeed with no PCF, neither the ‘Layer 1’ nor ‘Layer 2’ filtering removed the antibodies with TAP = −110. Similarly, as the ‘Layer 1’ stringency was increased, there was very little effect on the minimum, or the mean, TAP score. This is, perhaps, not surprising since the physicochemical properties on which this preliminary filtering is performed are somewhat similar to those exploited by the TAP score. However, the number of negative TAP scores does decrease as the ‘Layer 1’ filtering becomes more stringent (Table 4).

Again, because of the recalculation of the Z-scores and ellipse, there is one case in which the mean TAP score does not steadily progress closer to zero as the ‘Layer 1’ stringency is increased (Table 4, physicochemical filtering, Z-score = 0.5).

It is also interesting to observe that, comparing the output of ‘Layer 1’ and ‘Layer 2’, the minimum and mean TAP scores improve. Given that ‘Layer 2 is predicting clinical success rather than developability, there is no reason to expect that this would be the case. Indeed, the percentage of antibodies with negative TAP scores retained after ‘Layer 2’ is larger than that after ‘Layer 1’, indicating that ‘Layer 2’ filtering is indeed detecting something different from developability.

## Discussion

We have demonstrated the ability to triage library antibodies to find those with properties similar to currently available therapeutic mAbs. This was achieved through a combination of preliminary filtering using physicochemical properties (to remove clearly outlying mAbs), with unsupervised and supervised machine learning. This demonstrates a useful tool in monoclonal antibody therapeutic discovery that may be applied to new and preexisting paired human antibody libraries to identify possible clinical candidates with potential to pass clinical trials in order to avoid expensive late-stage failures. Parameters of the pipeline at each step may be adjusted such that increased or reduced stringency filtering can produce a smaller (but more likely to be successful) or larger selection of antibodies. This pipeline can be used to identify antibodies with properties of therapeutic mAbs from large libraries,^60,61^ to screen antibodies from transgenic animals following immunizations,^62^ or from human patients recovering from a condition of interest.^5^ Using the pipeline in these contexts reduces the experimental work in finding an antibody which has properties suitable for use in the clinic.^27^

The ‘Layer 1’ triage creates a 2D projection of the *N*-dimensional space resulting from the protein language model encoding. We found that all the clinical stage antibodies clustered in this space, and that the held-back set also clustered with the original set of clinical antibodies. Consequently, this must represent a region of the *N*-dimensional space that shares some properties and therefore other antibodies that are found in this region must have similar features. Nonetheless, it is perfectly possible that some antibodies that do not have suitable developability properties for use in the clinic may also fall within the bounds that we set around the cluster; it is also likely that some future clinically successful antibodies may not cluster well with the existing clinical set. The purpose of this step is to identify candidates with the highest chance of having good developability characteristics.

The pipeline allows for further optional triaging to be added at any point to give additional layers of stringency. The advantage of using these steps is a vastly reduced computation time. On an A5000 GPU used in this study, the AntiBERTy encoding takes 0.06 seconds per V_H_ and V_L_ pair, making it suitable for the high-throughput analysis of libraries (compared with the approximately 30 seconds required per antibody for the TAP Score web server^31^). While the protein language models may be doing so implicitly, using additional features also opens up the possibility of using other explicit features, including screening for immunogenicity^55^ and known sequence liabilities such as post-translation modification sites^60,63^ and hydrophobic patches.^24^

Direct comparisons of the performance of our method with the TAP score are not really possible. TAP relies on the distribution of a number of calculated and predicted physicochemical properties, some of which rely on a (predicted) structure of the antibody. While predicted properties can be compared with available experimental data, we only use physicochemical properties (calculated solely from sequence) as a preliminary screen to remove obvious outliers. The machine learning stages are based on a protein language model encoding that projects information (including implied structural features) into a high-dimensional space, which is then reduced to a 2D space in which clinical antibodies are seen to cluster. Consequently, we do not directly predict properties related to developability and comparisons with published experimental data are not possible.

It is also worth noting that our approach is not simply suggesting that if the sequences are more similar to those of clinical-stage antibodies, they should have better developability. If that were the case, we could just have used a BLAST search. Rather, we exploit the encoding from the antibody-specific protein language model, AntiBERTy and it is well established that protein language model encoding of sequences relates a sequence to structural and lineage information on which it has been trained and thus captures other key information. These encodings are highly sensitive and can even predict the effect of single amino acid changes.^64^ We have identified a 2D projection of the AntiBERTy encoding that clusters the clinical-stage antibodies and consequently we are looking at similarity of the protein language model encodings in those two principal components rather than sequence similarity *per se*.

Because human clinical antibodies clustered so closely in the kernel PCA, they must have similar features which have been encoded and recognized by the language model. The fact that the clinical antibodies cluster near the origin suggests that they are developable largely because they are ‘ordinary’ antibodies which innately satisfy the required conditions. As stated above, it is entirely possible that antibodies with very different properties could have therapeutic potential, but ultimately these would be higher-risk and consequently it is generally better to allow false negatives than false positives.

It is interesting to note that, using the TAP score, more than 25% of fully human clinical-stage antibodies exhibit negative TAP scores. Our approach clusters all the clinical antibodies and the ellipse function (with default parameters) will capture all of these, including those that have TAP red flags. In other words, if input antibodies are found to be located within the same region of the projection of the high-dimensional encoding, they are likely to have *sufficiently good* developability. We calculated the TAP scores for the 133 human clinical-stage antibodies and found that, while ~74% have a TAP score of zero (indicating no developability issues), the remainder have negative TAP scores as low as −30 (see Supplementary Figure S5).

While the unsupervised model (‘Layer 1’) groups together both approved and discontinued clinical antibodies, when these two groups are studied in a supervised context (‘Layer 2’), it is possible to recognize differences between them. Even though the dataset is small, the 10-fold cross-validation and held-back dataset demonstrate that there are features that are important for successfully completing clinical trials and, in future, larger datasets would allow us to have increased confidence in these predictions. The results also show that the light-chain Framework 3 seems to have a large contribution to these features. It is worth noting that this predictor is not identifying something trivial in the sequences. Supplementary Figure S2 shows that the distribution of germline light/heavy pairs is very similar in the antibodies that succeed or fail in clinical trials. As an additional control to look for simple sequence features, we also took a very simple approach of predicting that all antibodies with kappa light chains would succeed while all with lambda light chains would fail. As expected, this showed an MCC of 0.01 indicating no predictive power (data not shown).

A drug may be discontinued from trials for efficacy reasons relating to bioavailability or binding to the target, safety reasons relating to the antigen or antibody (including immunogenicity) as well as business or marketing reasons (including the existence of other good drugs).^1,9^ As discussed above, since we showed that there are no statistical differences between the approved and discontinued groups for thermostability, pI or CDR-H3 properties, it is possible that the model is selecting features related to immunogenicity, or V_H_/V_L_ germline gene pairing which may be related to stability.^65^ The latter option could then be related to biases seen in the approved and discontinued datasets perpetuated by the lead candidate selection processes, although the approved and discontinued dataset have similar proportions of V_H_/V_L_ germline gene pairing (Supplementary Figure S2), indicating other factors are being recognized in this region which are related to clinical trial success.

To summarize, we have developed a tool with a goal similar to methods such as TAP,^31^ but that works in a different way, exploiting ‘big data’ for protein language model encodings and exploring a large sample of the human repertoire, together with artificial intelligence. This work has demonstrated the ability to triage a library of antibodies to identify those with features similar to approved mAb therapeutics (and rejecting those that are very different) using language model encoding and applying them to both unsupervised and supervised machine learning. Furthermore, we demonstrate the ability to fine-tune the output in terms of quality by adjusting the thresholds of the models used to obtain the output. These tools aim to make use of previously curated and future antibody datasets to triage large datasets, enabling faster and cheaper identification of potential lead candidates.

## Methods

### Data collection

#### Human clinical-stage mAbs

Paired V_H_ and V_L_ sequences of therapeutic monoclonal antibodies (*n=*801) were downloaded from the October 2021 release of TheraSabDab.^3^ Therapeutics marked as ‘Whole mAb’ were selected and identified as being fully human using the ‘−umab’ suffix excluding instances of ‘−zumab’ (humanized). Each therapeutic was checked for its source using the literature. This resulted in a dataset of 143 antibodies: approved mAbs (*n=*31); discontinued mAbs (*n=*77) and in trials (*n=*35) (Supplementary Table S1). A further independent test dataset of human-derived clinical mAbs was acquired (*n=*203) using the 2016 naming convention in which the source infix was removed from the name and the 2022 naming convention using ‘−tug’ for unmodified whole immunoglobulins and ‘−bart’ for whole immunoglobulins with engineered amino acid changes in the constant domains^54^ (Supplementary Table S4).

#### Library antibodies from OAS

The Observed Antibody Space database^18^ was accessed in January 2022 and 34 libraries were downloaded totaling 88,274 paired sequences. A total of 10000 antibodies were selected randomly in order to create a training set for unsupervised learning (Suplementary Table S2).

#### Approved and discontinued mAbs

Clinical mAbs were obtained from the October 2021 release of the TheraSabDab database.^3^ The V_H_ and V_L_ sequences of 115 approved antibody drugs and 156 discontinued drugs were collected. Seven drugs were excluded from the discontinued dataset as they were found to be discontinued for reasons not related to efficacy or safety. Edrecolomab was also moved from the approved dataset and the discontinued dataset because it was later withdrawn for efficacy reasons.^66^ The result of this is a dataset of 115 approved and 150 discontinued antibodies (Supplementary Table S5). Excluded sequences and reasons for their exclusion are found in Supplementary Table S12. A held-back dataset of 21 therapeutics was taken from TheraSabDab accessed in October 2023 and not included in the original dataset (Supplementary Table S11).

### Test BCR dataset

The Test B cell receptor (BCR) sequence dataset^67^ used to demonstrate the pipeline was downloaded from dx.doi.org/10.5281/zenodo.5146019. This dataset was obtained from six healthy blood donors whose B cells were isolated and sorted via fluorescence-activated cell sorting by developmental stage. Transcripts from each individual cell were bar-coded making V_H_/V_L_ pairing possible. Antibody V_H_ and V_L_ pairs were taken from B cells which shared the same bar-code where both an IGH and IGλ or IGκ chain was present. In cases where both IGλ and IGκ chains were present, the chain with the highest count number was taken as the V_L_ chain pair. No filtering based on the type or BCR developmental stage was performed. Individual amino acid sequences for frameworks and complementarity-determining region (CDR) loops were concatenated to give the full antibody variable domain sequence. In total, 10,382 paired antibodies were extracted.

### Encoding H and L sequences with antibody language models

V_H_ and V_L_ sequences were numbered according to the Chothia scheme^68^ using AbNum^58^ (www.bioinf.org.uk/abs/abnum/.), where missing residues in the numbering scheme sequence were padded with characters dependent on which protein language model was being used, to align all sequences making V_H_ sequences 132 residues long and V_L_ sequences 122 residues long. Details of sequence encodings can be found in Table 5.

**Table 5.** Details of language model encodings.

Language Model	Features (VH+VL)	Padding Character	Reference
AntiBERTy	130,048	‘_’	50
AbLang	195,072	‘*’	18
Sapiens	152,560	‘*’	49
ESM (esm2 t6 8 M UR50D)	82,560	‘X’	44

### Supervised machine learning

Supervised learning was performed with SciKitLearn using 15 classifiers^69^ given in Table 6. Descriptions of each classifier used and details can be found in Supplementary File: Supplementary_ML.pdf.

**Table 6.** Supervised machine learning classifiers used in classifying approved and discontinued antibodies.

	Classifier	Acronym	Implementation
	Decision Tree		sklearn.tree.DecisionTreeClassifier
	Stochastic Gradient Descent Classifier	SGDC	sklearn.linear_model.SGDClassifier
	Ridge Classifier		sklearn.linear_model.RidgeClassifier
	Ridge Classifier CV		sklearn.linear_model.RidgeClassifierCV
	AdaBoost Classifier		sklearn.ensemble.GradientBoostingClassifier
	Gradient Boost Classifier		sklearn.ensemble.GradientBoostingClassifier
	Bagging Classifier		sklearn.ensemble.BaggingClassifier
	Random Forest Classifier		sklearn.ensemble.RandomForestClassifier
	Calibrated Classifier		sklearn.calibration.CalibratedClassifier
	Gaussian Naive Bayes Classifier	GaussianNB	sklearn.naive_bayes.GaussianNB
	Support Vector Machine	SVC	sklearn.svm.SVC
	Linear Support Vector Machine Classifier	LinearSVC	sklearn.svm.LinearSVC
	Logistic Regression Classifier		sklearn.linear_model.LogisticRegression
	Logistic Regression CV Classifier		sklearn.linear_model.LogisticRegressionCV
	Quadratic Discriminant Analysis Classifier	QDA	sklearn.discriminant_analysis.QuadraticDiscriminantAnalysis

F-regression is a method of feature reduction where the *k* most informative features are kept as input to the model. This is done by calculating the cross-correlation of each data point and the label for all features, which is converted to an F-score, then to a p-value and ranked.^70^ F-regression was implemented through the module sklearn.feature_selection.SelectKBest using the Python module sklearn.feature_selection.f_regression as the score function and variable numbers for *k* were substituted [1,10,50,100,500,1000,2500,5000,10000].

Once the F-regression was implemented on the encoded dataset, it was then split into training and test sets using sklearn.model_selection.train_test_split where training portions were used to train the models using ten-fold cross-validation.

Model performance was measured using the Matthews’ Correlation Coefficient (MCC),^57^ which gives a score between −1 (perfect inverse prediction) and 1 (perfect prediction), with 0 being random chance. Mean MCC and standard deviation for prediction performance over the ten folds were reported.

### Unsupervised machine learning

PCA was used as a method of dimensionality reduction and implemented through sklearn.decomposition.PCA. Non-linear (kernel) PCA^51^ was implemented through sklearn.decomposition.KernelPCA using kernel functions ‘rbf’, ‘cosine’ and ‘poly’ and two principal components. At first the coefficient of the kernel (γ) was set to the default value of *1/k* where *k* is the number of features. Once rbf was selected as the most suitable method, differing values for γ were tested [10, 50,100, 500, 1000]. t-distributed Stochastic Neighbor Embedding (t-SNE)^52^ was implemented through sklearn.manifold.TSNE with two components where the learning rate was set to 10, and the perplexity set to 1000. Uniform manifold approximation and projection (UMAP)^53^ was implemented through sklearn.manifold.UMAP with the learning rate set to 1 and the nearest neighbors set to 100.

### Ellipse function

The ellipse function takes in the points of the two extremes on the major axis (*x1, y1*) and (*x2, y2*), as well as a value for *h* (the height of the minor axis). The major axis is taken as the principal component where clinical mAbs have the largest distribution, and the selected points are given as the points on the distribution closest to a given Z-score in that distribution. The value of *h* is given as the distance between the two equivalent points on the minor axis. The method for producing the ellipse works as follows:

- Calculate the major and minor radii of the ellipse (*a* and *b*, respectively). The major radius is calculated from the two given points (Equation 1) and the minor radius is calculated as half the value given for *h*, where *Δx* is the difference in *x* values and *Δy* is the difference in *y* values between the two extreme points on the major axis.
  (1) $$a=\frac{\sqrt{\Delta x^{2}+\Delta y^{2}}}{2},b=\frac{h}{2}$$
- Use the parametric equation of an ellipse to generate the ellipse over 100 equally spaced points between 0 and 2π assuming it is centered at the origin (Equation 2). For a given point on the ellipse:
  (2) x=acos(θ),y=bsin(θ)

where *a* is the major axis radius, *b* is the minor axis radius and *θ* is a given angle between 0 and 2π.

- Calculate the angle between given points to obtain an angle of rotation using the Python Numpy arctan2 function^71^ for *Δy* and *Δx*.
- Calculate a rotation matrix (R) based on the angle of rotation:
  (3) $$R=\left[\left[\cos\left(\theta \right),-\sin\left(\theta \right)\right],\left[\sin\left(\theta \right),\cos\left(\theta \right)\right]\right]$$

where *θ* is the angle of rotation.

- Apply the rotation matrix R, to the ellipse.
- Calculate the midpoint of the two given points:
  (4) $$x=\frac{x_{1}+x_{2}}{2},y=\frac{y_{1}+y_{2}}{2}$$
- Translate the ellipse to the midpoint.
- For each point, check if its *x* and *y* coordinates are inside the ellipse using the Polygon function from the Python ‘Shapely’ module.

### Calculating physicochemical properties

Physicochemical properties were calculated as described below and compared between groups using the two-tailed unpaired Mann–Whitney U-test.^72^

#### Identifying CDR-H3 loops

The CDR loop three of the V_H_ domain (CDR-H3) has frequently been observed to have the largest contribution to antibody binding affinity because it is the most diverse region between sequences, overlapping the Variable, Diversity and Junction gene segments.^47,73^ CDR-H3 regions were identified using the AbNum software^58^ and applying the Kabat/Chothia/Martin definition (H95-H102). Sequences with more than two cysteine residues were excluded as additional cysteines are a known risk factor for aggregation.^74^

#### Thermostability

Gibbs Free Energy (*ΔG*) of unfolding was predicted for each antibody sequence using the Oobatake method^45^ with experimental values of *ΔH* and *ΔS* taken from the original paper. mAbs with negative *ΔG* of unfolding values were considered unstable and associated with poor developability. This was calculated for the V_H_ and V_L_ chains, as well as for both chains concatenated together using the ‘ssbio’ Python module.^75^

#### Isoelectric point

The method of calculating Isoelectric Point (pI) was that used in the IPC software^46^ which uses experimentally obtained peptide pKa values from the EMBOSS database^76^ substituted into a rearranged Henderson-Hasselbach equation. The equations are iterated using different pH values, starting at 6.5, and the results of the termini and each of the charged residues are summed together. If the sum is 0 ± 0.01, the isoelectric point is reached. Otherwise, the iteration continues to increase the pH if the summed net charge was positive or to decrease the pH if it was negative.

#### Immunogenicity (humanness)

The G score^56^ is a measure of antibody humanness based on similarity to germline families and a predictor of immunogenicity. This metric was calculated using the online tool www.bioinf.org.uk/abs/gscore/ for V_H_ and V_L_ independently. The minimum score of these chains for each antibody was taken and the mean for each of these sets of minima is presented.

#### V-region germline gene identification

V-region Germline genes were identified using the in-house ‘Assign GermLine’ software (AGL; github.com/AndrewCRMartin/agl/). Where more than one germline gene has the same (highest) sequence identity, AGL selects a gene using the logic that the germline family with the lowest family number was likely to have been discovered first and therefore likely to be more numerous. The same logic is applied to allelic variants and proximal genes are favored over distal genes, ensuring that gene names are consistent.

### TAP scores

TAP scores were developed by Raybould *et al*.^31^ to compare an antibody with the clinical dataset using metrics related to developability, assigning ‘amber penalties’ to antibodies that fall in the top and bottom 5% of the observed distribution, and ‘red flags’ to antibodies that fall outside the distribution. TAP scores were calculated for 10,382 paired V_H_ and V_L_ nucleotide sequences from the Test BCR dataset in batches of 500 using the IGX platform igx.bio/ in August 2023 using the default penalty set. Details of statistics measured and penalties assigned can be found in Raybould *et al*.^31^

## Supplementary Material

Supplementary_Figures.docx

Supplementary_ML.docx

Supplementary_Tables.xlsx
