# Supplementary material for: An antibody developability triaging pipeline exploiting protein language models
Source: MAbs. 2025 Mar 4;17(1):2472009. doi: 10.1080/19420862.2025.2472009 (PMC11901365; doi:10.1080/19420862.2025.2472009)
Supplement: Supplementary_Figures.docx [file KMAB_A_2472009_SM7940.docx]

# An Antibody Developability Triaging Pipeline Exploiting Protein Language Models

James Sweet-Jones and Andrew C.R. Martin

# Supplementary Figures


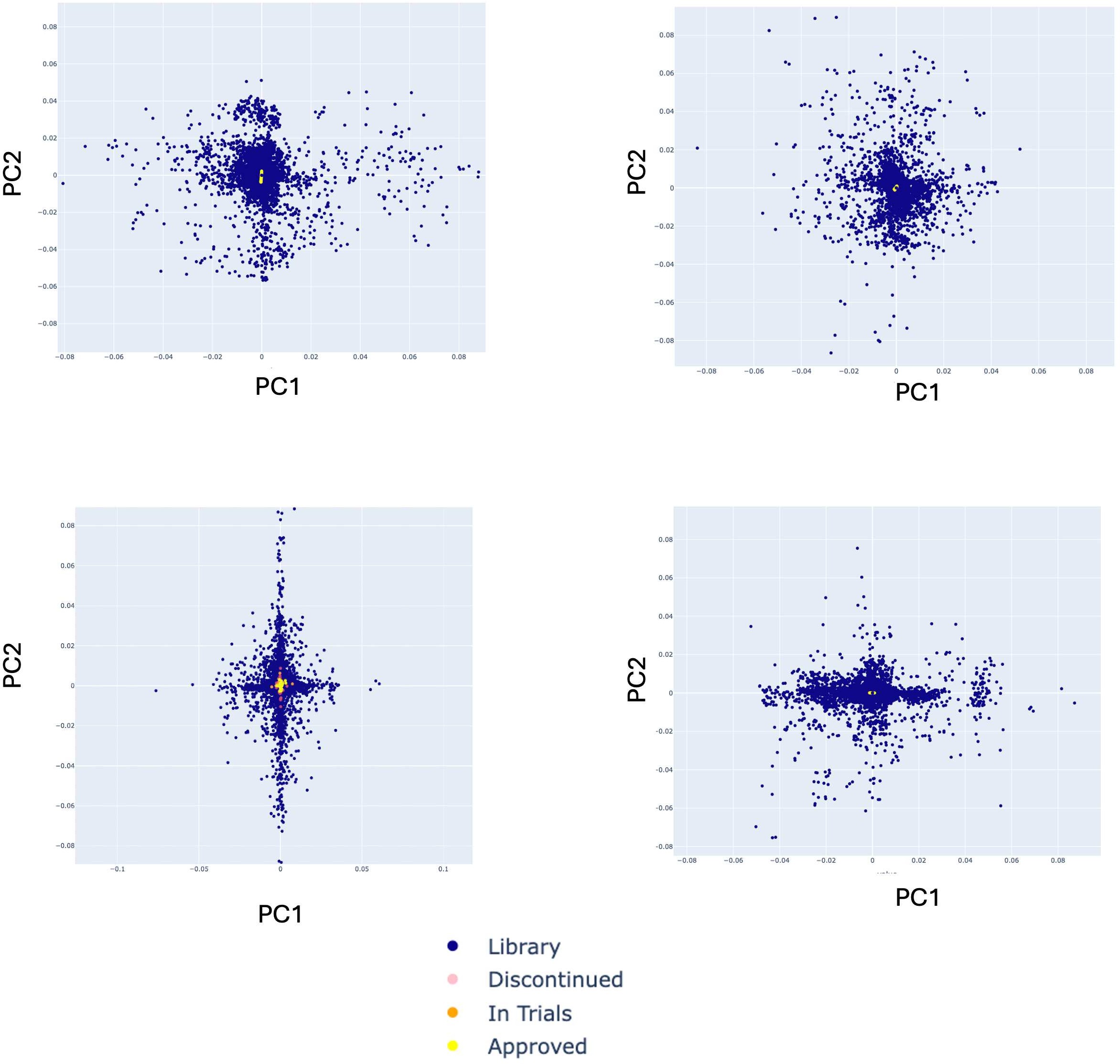


Supplementary Figure S1: Scatter plots of clinical and library datasets en- coded with the AntiBERTy language model and having undergone dimension- ality reduction using Kernel Principal Component Analysis with a radial basis function kernel (rbf). Different values for the coefficient of the kernel function (*γ* = [10*,* 100*,* 500*,* 1000]) are shown.

Alt-text: Four scatter plots demonstrating how different values of the kernel coefficient affect the shape of the Kernel PCA plot produced. The best overall clustering is seen with a kernel coefficient of 500 where the library antibodies are mostly spread along the two principal components and the clinical antibodies (discontinued, in-trials and approved) are all clustered at the centre of the plot.


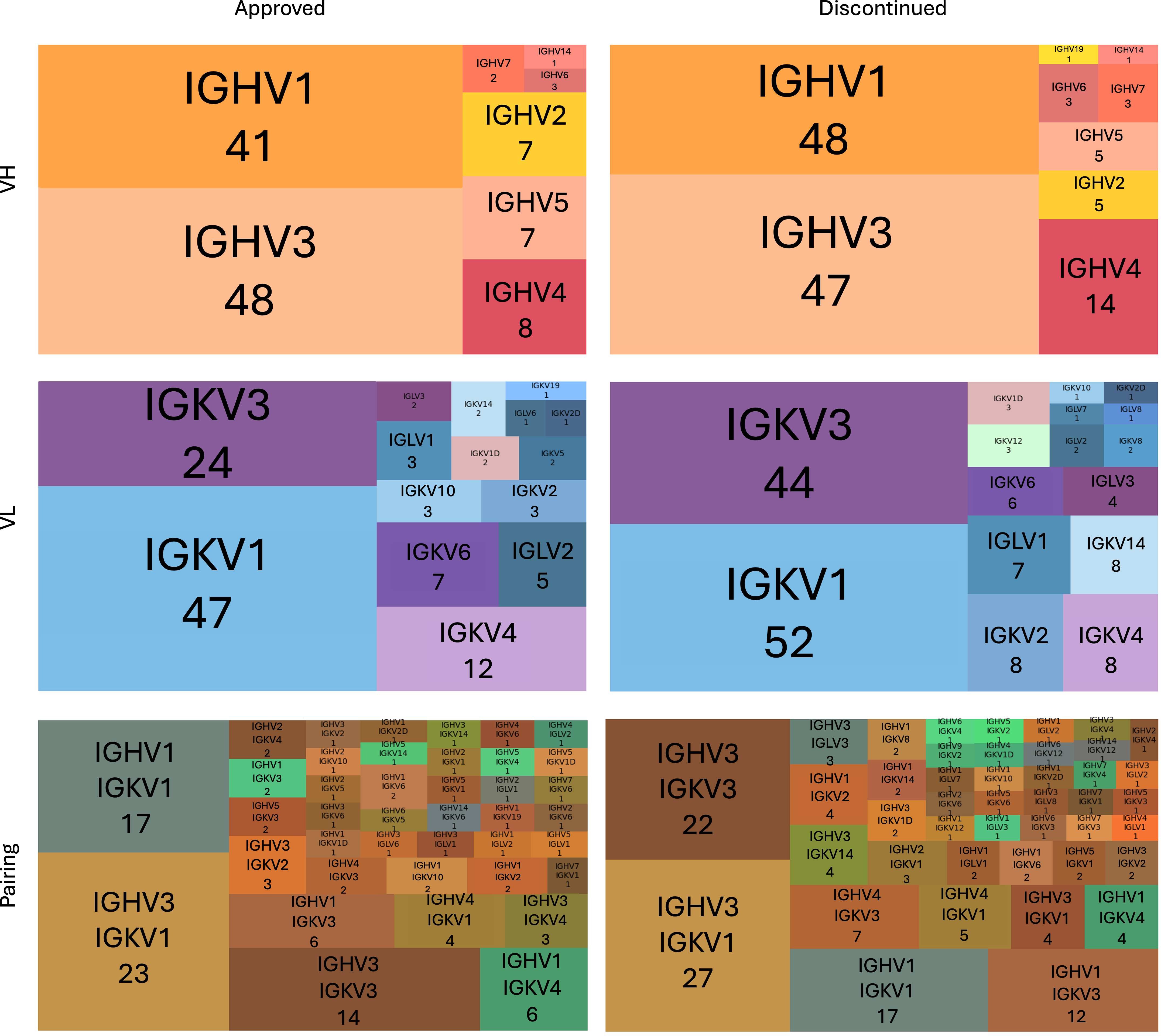


Supplementary Figure S2: Approved and discontinued germline pairing propor- tions. Proportions of germline gene families in approved (a) and discontinued

(b) antibodies. Colour coding for VH, VL and Pairing categories are consistent for approved and discontinued figures. This demonstrates that the germline us- age is similar in approved and discontinued groups and that these had broadly similar proportions of VH/VL/ V-gene germline pairings.

Alt-text: Two rows of three pie charts where the top row represents ap- proved, and the bottom represents discontinued, clinical antibodies. For each row, from left to right, the pie charts give the proportions of different V-region gene germlines for VH sequences, VL sequences and the pairings of VH and VL germlines respectively. For both rows (approved and discontinued), the figure shows that the distributions are broadly similar with large proportions of IGHV3 and IGHV1 in VH sequences, and IGKV1 and IGKV3 in VL sequences.


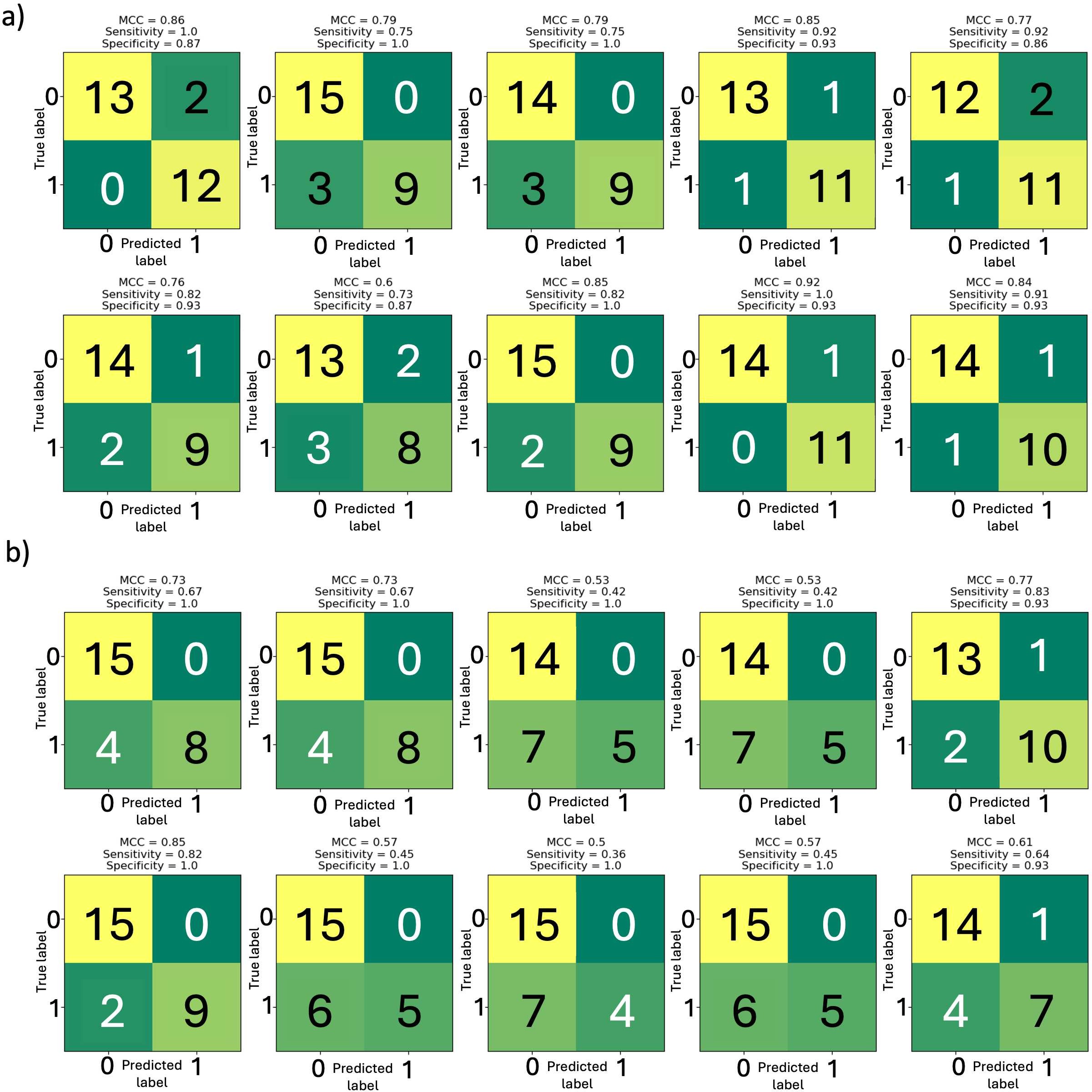


Supplementary Figure S3: Confusion matrices for each of the 10 splits during 10-fold cross-validation of the dataset of approved (class 1) and discontinued (class 0) mAbs encoded with the AntiBERTy language model and trained using LinearSVC showing when probability of taking a positive result was given as

a) 0.5 or b) 0.8.

Alt-text: Parts a) and b) each show two rows of 5 confusion matrices with true labels on the y-axis and predicted labels on the x-axis. The 10 confusion matrices in each part result from the 10 folds of cross-validation. For each confusion matrix, the MCC, Sensitivity and Specificity are shown. Part a) is for a prediction threshold of 0.5 while Part b) is for a prediction threshold of 0.8.


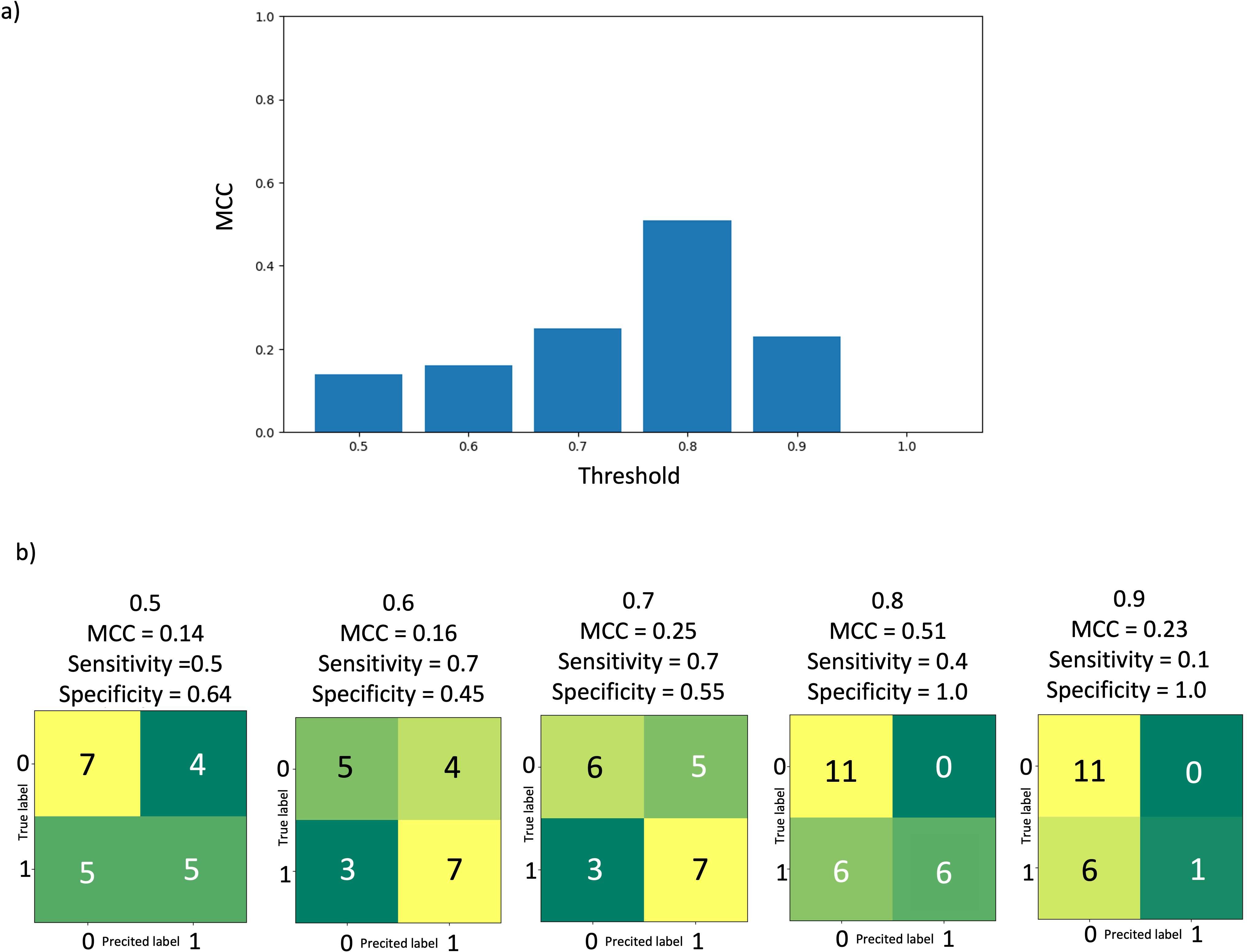


Supplementary Figure S4: a) Matthews’ Correlation Coefficient for the held- back dataset of 10 approved and 11 discontinued therapeutic antibodies at pre- diction thresholds of 0.5, 0.6, 0.7, 0.8 and 0.9. b) Confusion matrices, MCC scores, sensitivity and specificity for the held back data set at each of the pre- diction thresholds.

Alt-text: Part a) shows a bar chart of MCC scores obtained from the Lin- earSVC model trained using *k* = 2500 features from approved and discontinued antibodies and tested on a held-back dataset of approved and discontinued an- tibodies using increasing prediction thresholds (0.5 to 1.0 in steps of 0.1) for accepting a positive value. This demonstrates that a threshold of 0.8 gives the best performance. Part b) shows the 5 confusion matrices for the same predic- tion thresholds together with the MCC, Sensitivity and Specificity.


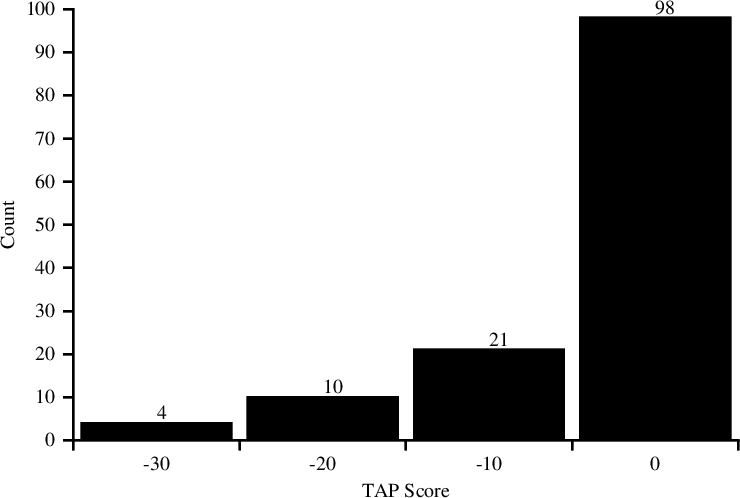


Supplementary Figure S5: Distribution of TAP scores for the 133 human clinical-stage antibodies showing that around 26% of these have a negative TAP score. The worst possible TAP score is −160 and our dataset of repertoire hu-

man sequence showed a worst score of −110 (although this was an exceptional

example).

Alt-text: A histogram of the count of human clincal-stage antibodies hav- ing different TAP scores: TAP=0, 98 examples; TAP=−10, 21 examples; TAP=−20, 10 examples; TAP=−30, 4 examples.
