# Supplementary material for: An antibody developability triaging pipeline exploiting protein language models
Source: MAbs. 2025 Mar 4;17(1):2472009. doi: 10.1080/19420862.2025.2472009 (PMC11901365; doi:10.1080/19420862.2025.2472009)
Supplement: Supplementary_ML.docx [file KMAB_A_2472009_SM7939.docx]

# An Antibody Developability Triaging Pipeline Exploiting Protein Language Models

James Sweet-Jones and Andrew C.R. Martin

# Supplementary Material: Supervised Learning Classifiers

## Decision Tree

Decision Trees are a methods of binary classification which are able to learn a set of rules as to how a data point should be classified using its features. These rules are established by finding a splitting point that minimises mean squared error for the new partitions [1]. Decision trees were implemented through from sklearn.tree.DecisionTreeClassifier with default parameters.

## Stochastic Gradient Descent Classifier

The Stochastic Gradient Descent (SGD) Classifier uses sampled data to improve gradient descent rather than using all of the data. SGD Classifier was imple- mented from sklearn.linear_model.SGDClassifier with default parameters.

## Ridge Classifier

A Ridge Classifier is a linear classifier model with penalty on large coefficients. The regularization encourages a more stable and generalisable model. The model minimizes an objective function combining a loss term and regularization, making it effective for handling correlated features. The Ridge Classifier was implemented through from sklearn.linear_model.RidgeClassifier with de- fault parameters.

## Ridge Classifier CV

Similar to above but performs leave-one-out cross-validation and implemented with sklearn.linear_model.RidgeClassifierCV

## AdaBoost Classifier

Adaptive Boosting works on similar principles to Gradient Boosting but here, the model continues to correct previous iterations by placing more attention to underfitted results of the previous model. AdaBoost was implemented through sklearn.ensemble.GradientBoostingClassifier with default parameters.

## Gradient Boost Classifier

Gradient Boosting Classification is performed by adding models sequentially to an ensemble of predictors so that the combined loss of all models is lower than the previous. Unlike Random Forests, this model uses gradient descent, which is an optimization technique to minimise the loss function. Gradient Boosting was implemented through sklearn.ensemble.GradientBoostingClassifier with default parameters.

## Bagging Classifier

“Bagging” Classifier also works similarly by combining weak learners where their voted or mean score forms a strong ensemble through random sampling with replacement from the dataset. A Bagging Classifier was implemented through sklearn.ensemble.BaggingClassifier with default parameters.

## Random Forest Classifier

Forests are a collection of decision trees trained on bootstrapping samples of a training dataset with replacement and additionally, a random sampling of the features of that dataset. The result of a set of input data points are averaged across the collection to overcome the overfitting problem usually encountered in decision trees through a user-curated ensemble of trees where the number of trees and pruning of trees can be tuned [1]. A Random Forest was im- plemented through sklearn.ensemble.RandomForestClassifier with default parameters.

## Calibrated Classifier

Calibrated Classifier aims to reflect the probability of an outcome to a class and to measure the confidence of that prediction. Unlike other classifiers that output a probability to assign classes, the probabilities are calibrated according to the frequencies of each class in the dataset. The Calibrated Classifier was implemented through sklearn.calibration.CalibratedClassifier with de- fault parameters.

## Gaussian Na¨ıve Baye Classifier

Gaussian Na¨ıve Bayes follows an assumption that each feature has an inde- pendent ability to predict an output label, and seeks to combine all of these

predictive powers into the final model. It also assumes all features follow a normal distribution and that Bayes’ Theorem of conditional probability can be applied to each feature by the way of a probability density function. Gaussian NB was implemented through sklearn.naive_bayes.GaussianNB with default parameters.

## Support Vector Machine Classifier

Support Vector Machine Classifier (SVC) work by representing a highly dimen- sional dataset in 2 dimensional space using dimensionality reduction and works to separate the two classes of labelled data using a kernel. Using the data points of class 0 and class 1 that are the physically closest to the hyperplane, the sup- port vectors, the marginal space is maximised between the two classes [2] using a special loss function called the Hinge Loss. SVC was implemented through sklearn.svm.SVC with default parameters.

## Linear Support Vector Machine Classifier

LinearSVC is a similar algorithm to SVC but minimises the squared hinge loss and penalises the size of bias by setting a linear kernel rather than a polyno- mial one. LinearSVC was implemented through sklearn.svm.LinearSVC with default parameters.

## Logistic Regression Classifier

A linear model that can be applied to binary classification problems by calcu- lating a predicted probability using a combination of features from an input datapoint and assigning an input data point to a class using a threshold value typically set at 0.5. Logistic Regression Classifier was implemented through sklearn.linear_model.LogisticRegression with default parameters.

## Logistic Regression CV Classifier

A linear model that can be applied to binary classification problems by calculat- ing a predicted probability using a combination of features from an input dat- apoint and assigning an inputted data point to a class using a threshold value typically set at 0.5. Logistic Regression Classifier was implemented through sklearn.linear_model.LogisticRegressionCV with default parameters.

## Quadratic Discriminant Analysis Classifier

Like Linear Discriminant Analysis, Quadratic Discriminant Analysis aims to classify samples using conditional probabilities, however, using quadratic equations allows the use of a curved hyperplane to separate datapoints. This model was implemented through sklearn.discriminant_analysis.QuadraticDiscriminantAnalysis with default parameters

## References

1. Danielle Denisko and Michael M Hoffman. Classification and interac- tion in random forests. *Proceedings of the National Academy of Sciences*, 115(8):1690–1692, 2018.
2. Asa Ben-Hur, Cheng Soon Ong, S¨oren Sonnenburg, Bernhard Sch¨olkopf, and Gunnar R¨atsch. Support vector machines and kernels for computational biology. *PLOS Computational Biology*, 4(10):e1000173, 2008.
